# Type I interferon-mediated autoinflammation in two unrelated patients due to a proximal intronic splice site variant in *DNASE2*

**DOI:** 10.21203/rs.3.rs-7039031/v1

**Published:** 2025-07-31

**Authors:** Oskar Schnappauf, Hongying Wang, Qing Zhou, Vasily Burlakov, Anna Kozlova, David B Beck, Natalia Sampaio Moura, Natalie T Deuitch, Wanxia Li Tsai, Massimo Gadina, Patrycja Hoffmann, Amanda K Ombrello, Victoria Zakharova, Amina Kieva, Elena Raikina, Federica Ricucci, Daniel L. Kastner, Anna Shcherbina, Ivona Aksentijevich

**Affiliations:** 1. Department of Natural Sciences & Institute for Functional Gene Analytics (IFGA), Bonn-Rhein-Sieg University of Applied Sciences, Rheinbach, Germany; 2. Inflammatory Disease Section, National Human Genome Research Institute, Bethesda, Maryland, USA; 3. Liangzhu Laboratory, Zhejiang University, Hangzhou, China; 4. Center for Human Genetics and Genomics, New York University Grossman School of Medicine, New York, NY, USA; 5. National Institute of Arthritis and Musculoskeletal and Skin Diseases, Bethesda, Maryland, USA; 6. Dmitry Rogachev National Medical and Research Center for Pediatric Hematology, Oncology and Immunology, Moscow, Russia; 7. Ilyinskaya Hospital, Moscow, Russia

**Keywords:** Type I interferonopathy, DNASE2, Autoinflammation, Splice site variant, JAK-STAT signaling

## Abstract

**Purpose:**

Type I interferonopathies are Mendelian inborn errors of immunity caused by defective clearance or recognition of nucleic acids, resulting in chronic type I interferon (IFN) activation. DNase II is a lysosomal endonuclease critical for degrading DNA during erythropoiesis and apoptosis. Biallelic loss-of-function variants in *DNASE2* cause a rare autoinflammatory disorder. We aimed to characterize the clinical, genetic, and functional impact of a novel *DNASE2* splice site variant in two unrelated patients.

**Methods:**

We performed exome and RNA sequencing to identify and validate variants. A minigene assay assessed splicing effects. DNase II activity was tested in cell lysates. Structural modeling, cytokine profiling, and immune stimulation assays were conducted to evaluate pathway activation and therapeutic response.

**Results:**

Both patients carried a homozygous *DNASE2* splice site variant (c.511+5G>A), resulting in exon 4 skipping and impaired DNase II activity. They presented at birth with rash, thrombocytopenia, and anemia, later developing neutropenia, arthritis, failure to thrive, and systemic inflammation. One patient also harbored a homozygous *USP43* variant that reduced USP43 expression and enhanced IFN signaling. Both patients showed increased JAK-STAT activation and responded clinically to JAK1/2 inhibition with ruxolitinib.

**Conclusion:**

We report a novel proximal intronic splice variant in *DNASE2* associated with DNase II deficiency and type I interferon–mediated autoinflammation. Our findings expand the genotypic and phenotypic spectrum of this disorder, highlight the role of intronic variants in rare immune diseases, and support JAK inhibition as a targeted treatment approach.

## Introduction

Systemic autoinflammatory diseases (SAIDs) encompass a heterogeneous group of disorders characterized by dysregulated innate immune responses, manifesting in recurrent episodes of sterile inflammation [1,2]. These conditions impose substantial morbidity, often resulting in chronic, debilitating pain, and progressive organ damage, underscoring the urgent need for detailed mechanistic insights and novel therapeutic targets. A common feature in many SAIDs is the perturbation of cellular signaling pathways involving nucleic acid metabolism, which is pivotal in immune regulation [3]. Dysregulated nucleic acid metabolism and sensing can precipitate an exaggerated interferon (IFN) response through the JAK-STAT pathway, effectively mimicking a viral infection at the cellular level [4]. The clinical significance of aberrant nucleic acid metabolism is exemplified by Mendelian type I interferonopathies, such as DNase II deficiency, first described in 2017 [5].

DNase II is a lysosomal endonuclease predominantly expressed in macrophages, where it degrades nuclear DNA from apoptotic cells and enucleated erythroid precursors [6]. In murine models, DNase II deficiency is embryonically lethal due to the accumulation of undigested DNA, which activates cytosolic DNA sensors and leads to excessive cytokine production, including type I IFNs [7]. Notably, this lethal phenotype can be mitigated in Dnase II-deficient mice crossed with *Sting* or *cGas* mutants, underscoring the critical role of Toll-like receptor (TLR)-independent cytosolic DNA sensing pathways in driving inflammation [7,8]. In humans, biallelic hypomorphic or LoF variants in *DNASE2* cause a rare clinical syndrome marked by neonatal anemia, a prominent IFN signature, nephropathy, and arthritis [5,9-11].

Beyond nucleic acid metabolism, protein ubiquitination is essential for modulating immune responses [12]. Ubiquitination, a post-translational modification, influences protein activity, subcellular localization, and stability, with reversibility conferred by deubiquitinases (DUBs). Dysregulation of DUBs has been implicated in various inherited and acquired autoinflammatory syndromes [13]. For example, haploinsufficiency of A20 results in uncontrolled nuclear factor-kappa B (NF-κB) signaling and early-onset systemic inflammation, while biallelic hypomorphic mutations in *OTULIN* cause severe autoinflammatory disease [14-18]. USP43, or ubiquitin-specific peptidase 43, is a DUB that has been shown to play a role in cancer pathogenesis and potentially in antiviral innate immunity. USP43 has also been identified as a regulator of HIF1 signaling and NF-kB signaling through IκBα degradation [19,20].

In this study, we report two unrelated patients with DNase II deficiency, both exhibiting marked inflammatory phenotypes, growth failure, subcutaneous nodules, osteopenia, severe arthritis with contractures, and hepatosplenomegaly. Genetic analyses revealed a novel homozygous pathogenic variant in *DNASE2* in both cases, confirming DNase II deficiency as the causative defect. Additionally, one patient carried a homozygous LoF variant in *USP43*, potentially exacerbating the clinical phenotype via dysregulated ubiquitination and amplified IFN signaling. This unique combination of genetic mutations may elucidate the heightened inflammatory presentation in this patient.

## Methods

### a. Ethic Statement

Written informed consent was obtained from the patients, their legal guardians, and family members in accordance with the Declaration of Helsinki. The study was approved by the Institutional Review Board of the National Human Genome Research Institute.

### b. Sample Collection and PBMC Isolation

Peripheral blood mononuclear cells (PBMCs) were isolated from whole blood using Ficoll-Paque PLUS (GE Healthcare) density-gradient centrifugation. Cells were cryopreserved in fetal bovine serum containing 10% DMSO and stored in liquid nitrogen. Serum and plasma were separated from coagulated and heparinized blood, aliquoted, and stored at −80°C. For RNA analyses, blood was collected into PAXgene Blood RNA Tubes (PreAnalytiX).

### c. Custom gene panel and Exome Sequencing

Genomic DNA was extracted from peripheral blood using the Maxwell^®^ 16 Blood DNA Purification Kit (Promega). Exome libraries were prepared using the Illumina TruSeq DNA Sample Preparation Kit and sequenced on an Illumina HiSeq2000. Reads were processed following GATK Best Practices, including base recalibration, indel realignment, and joint genotyping [21]. Variants were annotated using VEP and filtered for rare, predicted deleterious variants within coding regions and canonical splice sites. Prioritization was performed using GEMINI and SliVar [22,23]. For custom gene panel molecular genetic analysis see supplementary information.

### d. Sanger Sequencing

Genomic DNA samples were extracted from peripheral blood using the Maxwell^®^ 16 Blood DNA Purification Kit (Promega). Coding and flanking intronic region of exon 4 of *DNASE2* (RefSeq: NM_001375.3) were amplified using AmpliTaq Gold Fast PCR Master Mix (Thermo Fisher Scientific). Following primer pair with M13 sequencing tails was used: F: 5’-GTCCACCACCACGCTCAG*GGTCCAGGAGGTCAGAGACA*-3’ and R: 5’-AGGGTTCTTGGCTAACGTGG CTGAAGCCTCAGGTTCTTGC-3’. Sequencing of the forward and reverse strand was performed on a 3130xl Genetic Analyzer (Applied Biosystems) using manufactures settings. cDNA amplification and sequencing were performed accordingly using the following primer pair: F: 5’-TGCCAGCTCTTAGAGGGT- 3’ and R: 5’-AGGGTTCTTGGCTAACGTGGTG-3’. Data were analyzed with Sequencher.

### e. RNA Extraction and RT-PCR

Total RNA was isolated from blood (PAXgene Blood RNA Kit) and cultured cells (NEB Monarch RNA Miniprep Kit or TRIzol). cDNA was synthesized using SuperScript^™^ III Reverse Transcriptase. PCR was performed to assess splicing and expression of *DNASE2* using exon-flanking primers.

### f. RNA sequencing

Total RNA was isolated from whole blood collected in PAXgene Blood RNA Tubes using the PAXgene Blood RNA Kit (PreAnalytiX) as per the manufacturer's instructions, and from PBMCs using TRIzol (Thermo Fisher Scientific). High-quality RNA (RIN > 8) was used for cDNA library preparation with the TruSeq Stranded mRNA Library Preparation kit for NeoPrep (Illumina). Sequencing was conducted on an Illumina HiSeq 3000 System in 1 × 50 bp single read mode. Reads were mapped to the human reference genome (GRCh38) using TopHat v2.1.1. Gene expression (RPKM) was calculated using DeSeq2.

### g. Minigene Splicing Assay

A *DNASE2* minigene construct containing exon 4 and flanking intronic sequences was cloned into an expression vector, with wild-type and mutant (c.511+5G>A) alleles (GenScript). HEK293T cells were transfected with ViaFect^™^ (Promega), and cDNA was analyzed by RT-PCR and Sanger sequencing.

### h. Western Blotting and Immunoprecipitation

Cells were lysed in RIPA buffer. For USP43 complex detection, 3–8% Tris-Acetate gels were used. Other proteins were run on 4–12% gels. After transfer to membranes, blots were probed for DNASE2, USP43, pSTAT1, ISG15, LC3, actin, and Ub (K48/K63). Band density was analyzed using LI-COR Image Studio. For immunoprecipitation, lysates were incubated with anti-USP43 or anti-Ub antibodies and Protein A/G beads.

### i. Cell Stimulation and pSTAT1/pSTAT3 Flow Cytometry

PBMCs were stimulated with IFN-γ (100 ng/mL) or IL-6 (50 ng/mL) at 37°C for 15 min or 3 h. Cells were fixed in 4% paraformaldehyde, permeabilized with 90% methanol, and stained with lineage markers (CD14-PE, CD19-PE, CD3, CD4, CD8) and phospho-STAT antibodies (pSTAT1-Alexa Fluor 647, BD Pharmingen; pSTAT3-Alexa Fluor 647). Data were acquired using a BD FACSCanto and analyzed with FlowJo software.

### j. Nanostring IFN Signature

Expression of IFN-stimulated genes was assessed using a 32-gene Nanostring panel on total RNA (100 ng). Hybridization was performed on the nCounter Prep Station, and analysis was done using nSolver and MATLAB.

### k. Cytokine Profiling and ELISA

Serum cytokines were quantified using Bio-Plex Pro Human 37-plex and 27-plex kits (Bio-Rad). IL-6, IP-10, and MIG were measured in cell culture supernatants using ELISA kits (BioLegend, R&D Systems). Measurements were performed in technical duplicates or triplicates.

### l. USP43 Knockdown and Knockout

shRNA and CRISPR/Cas9 constructs targeting USP43 (SCBT) were electroporated into THP1 or HEK293T cells using a Neon transfection system (Invitrogen). Knockdown/knockout efficiency was confirmed by Western blot.

### m. Luciferase Reporter Assay

HEK293T USP43-KO and control cells were transfected with an IFN-stimulated response element (ISRE)-luciferase reporter. Cells were stimulated with IFN-α or IFN-γ for 24 hours, and luciferase activity was measured using a Dual-Luciferase Assay System (Promega).

### n. USP43 Rescue in Patient-Derived Cells

Flag-tagged USP43 plasmids were electroporated into patient-derived EBV-B cells followed by G418 selection. Expression and functional rescue were assessed by Western blot and cytokine assays.

### o. DNA Degradation Assay

Plasmid DNA (250 ng) was incubated with cell lysates in acidic buffer (30 mM NaAc, 20 mM EDTA, pH 5.2) at 37°C for 30 minutes. Reaction mixtures were analyzed on 1.2% agarose gels.

### p. Statistical Analysis

Data are presented as mean ± SD. Comparisons were evaluated using two-tailed paired t-tests or ANOVA as appropriate in Prism (GraphPad). p < 0.05 was considered statistically significant. Experiments were repeated independently at least three times unless otherwise noted.

## Results

### a. Clinical phenotype

**Fig. 1:**
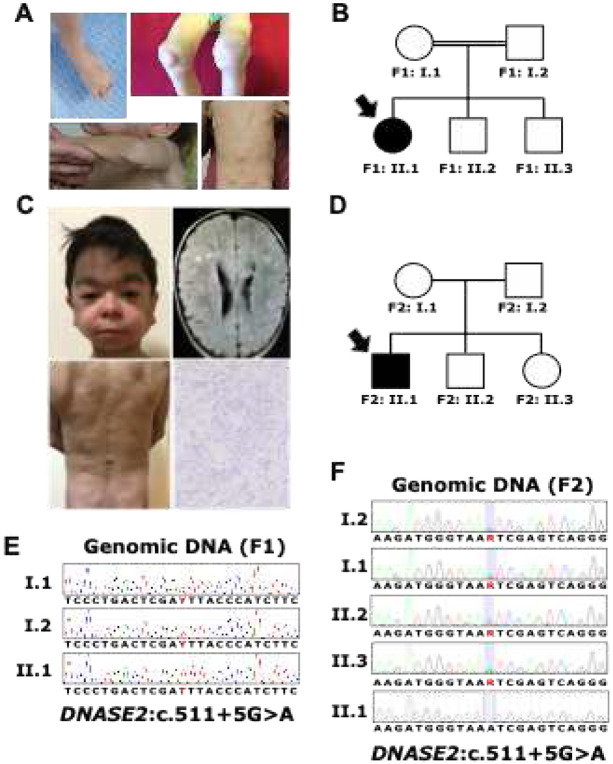
Patient presentation and *DNASE2* genotyping in the probands and their families (A) Clinical features of Patient P1 (F1: II.1), showing toes and knees arthritis and subcutaneous nodules. (B) Pedigree of Family 1, illustrating an autosomal recessive inheritance pattern with documented consanguinity, as indicated by the double line between the parents. (C) Clinical presentation of Patient P2 (F2: II.1), including facial lypodystrophy, MRI images of white matter lesions, macro- and microscopic (H&E staining) pictures of subcutaneous nodules, demonstrating granulomatous inflammation. (D) Pedigree of Family 2, demonstrating a recessive inheritance pattern with no consanguinity reported. (E) Sanger sequencing confirmation of the *DNASE2* variant *DNASE2*:c.511+5G>A, p. ? in family 1 (F1), with the proband carrying a homozygous variant and both parents being heterozygous carriers. Genomic sequence depicted. (F) Sanger sequencing confirmation of the *DNASE2* variant in family 2 (F2). Sequencing traces are shown for both parents (I.1 and I.2) and three offspring (II.1, II.2, II.3), demonstrating that the proband (II.1) is homozygous for the variant, while the parents and unaffected siblings are heterozygous carriers. Coding sequence depicted

Patient 1 (P1), a female born in 2010 to first-cousin consanguineous parents of Tatar ancestry, was followed at the D. Rogachev Center and referred to the National Institutes of Health at age 5 for evaluation of an undiagnosed autoinflammatory syndrome. She presented with a history of hemorrhagic rash and thrombocytopenia at birth. At 16 months, she experienced a recurrence of symptoms, including rash, thrombocytopenia, epistaxis, oral ulcers, arthralgia, osteopenia, abdominal pain, and hepatosplenomegaly. Despite treatment with intravenous immunoglobulin and steroids, her symptoms persisted. By the age of 2, she developed recurrent fevers, worsening arthralgias, joint contractures, and nodular rashes, leading to extensive but inconclusive evaluations, including colonoscopy, esophagogastroduodenoscopy, skin biopsy and multiple lumbar punctures (Fig. 1A). She was diagnosed with failure to thrive. Otherwise, her developmental milestones were normal, and no gross neurological deficits have been observed. Previous evaluations, including infection and paraneoplastic workups, were negative. A skin biopsy revealed non-specific granulomatous inflammation, and a colonoscopy indicated non-specific colitis. Genetic testing for pathogenic variants in autoinflammatory disease-associated genes was negative. Because of suspected autoinflammatory disease the patient has been consecutively and unsuccessfully treated with steroids and methotrexate, tocilizumab, infliximab, etanercept, anakinra, canakinumab. Treatment with 5 mg/day tofacitinib reduced her skin manifestations but did not ameliorate other symptoms. At the age of 5 her condition exacerbated, with severe anemia and neutropenia, failure to thrive, progressive arthritis. She received four weekly infusions of rituximab 375 mg/m^2^ and was started on ruxolitinib 1 mg/kg per day with dramatic improvement of her symptoms and eventual complete remission after 3 years of treatment. Both parents and her two older male siblings are healthy (Fig. 1B).

Patient 2 (P2), a male born in 2010 to unrelated parents of Bashkir origin, presented at birth with hemolytic anemia requiring periodic transfusions until the age of 2, which improved with steroid treatment. From the age of 2, he experienced recurrent inflammatory episodes lasting several days, joint pain with mild arthritis, lipodystrophy, growth retardation and the development of subcutaneous nodules (Fig. 1C). Hepatosplenomegaly was noted since the age 5 and currently progressed to liver steatosis and fibrosis. His renal involvement included mild proteinuria, though no biopsy was performed. Notably, he has osteopenia, possibly secondary to prolonged steroid use, and has been developmentally normal. Lymphopenia, recurrent neutropenia, elevated antinuclear antibody (ANA) and antineutrophil cytoplasmic antibody (ANCA) levels, and high immunoglobulin (Ig) levels were documented. He was diagnosed with hypothyroidism, managed with hormone replacement. His inflammatory markers, including erythrocyte sedimentation rate (ESR) and C-reactive protein (CRP), were consistently elevated. He received low dose steroids and tofacitinib, with partial resolution of his symptoms. The patient was referred to D. Rogachev Center at the age of 11, received four weekly infusions of rituximab 375 mg/m^2^. Currently he is managed on combined therapy of ruxolitinib 0.8 mg/kg/day, and the IL-6 inhibitor tocilizumab, that allowed to wean him off steroids and has led to remission of fever, arthritis, kidney disease and stabilization of liver disease. The patient’s older siblings (male and female) are healthy (Fig. 1D). The clinical manifestations of both patients closely resemble the presentation of patients with DNase II deficiency described to date (see table S1 and Fig. S1).

### b. Identification of a rare homozygous splice site variant in *DNASE2* in both patients

To investigate a potential genetic cause for the patients’ manifestations, exome sequencing was conducted on P1 and P2. Variants with an allele frequency of less than 0.001% in gnomAD v4.1.0 database were considered, including all coding variants and those within ±6 base pairs of exon-intron boundaries. A homozygous proximal intronic splice site variant in the *DNASE2* gene was identified in both patients (NM_001375.3: c.511+5G>A). Splice site algorithms SpliceAI and Pangolin predicted a pathogenic effect on splicing due to loss of a splice donor site. The variant was observed at a frequency of 1.8×10^−5^ in the gnomAD v4.1.0 database with no homozygous occurrences identified. In the ClinVar-Database, the variant is classified as a variant of uncertain significance (ClinVar ID: 1463731). Sanger sequencing of the patients and their parents confirmed the correct segregation of the variant (Fig. 1E, F; S2).

### c. Functional characterization of the effect of variant *DNASE2*: c.511+5G>A on splicing

**Fig. 2:**
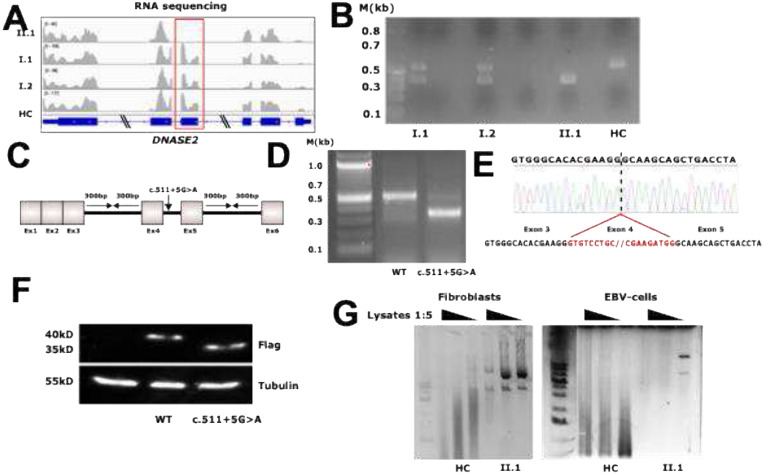
Functional characterization of the *DNASE2* c.511+5G>A, p.? splice variant (A) RNA sequencing analysis of *DNASE2* expression. Integrative Genomics Viewer (IGV) visualization of RNA-sequencing reads aligned to *DNASE2*, highlighting exon 4 with a red box. No read coverage in exon 4 suggests exon skipping in patient-derived RNA samples. (B) cDNA analysis of *DNASE2* transcript expression. Agarose gel electrophoresis of RT-PCR products from *DNASE2* cDNA amplified using flanking primers on RNA extracted from whole blood of the proband (II.1), both heterozygous carrier parents (I.1, I.2), and healthy controls (HC). The patient (II.1) shows an aberrant banding pattern indicative of exon skipping. (C) DNase II enzymatic activity assay. DNase II activity was assessed using an in vitro degradation assay of circularized plasmid DNA incubated with lysates from fibroblasts and Epstein-Barr virus (EBV)-transformed B cells derived from the patient (II.1), a healthy control (HC), and an unaffected sibling control (SC). A reduction in DNase II activity is evident in patient-derived cells compared to controls, suggesting functional impairment due to the splicing defect. (D) Minigene assay for splicing analysis. Schematic representation of the *DNASE2* minigene construct used to functionally assess the impact of the c.511+5G>A, p.? splice variant. (E) Splicing defect validation using the minigene construct. Agarose gel electrophoresis of RT-PCR products generated from HEK293T cells transfected with either the wild-type (WT) or mutant (c.511G>A) *DNASE2* minigene construct. The mutant construct shows an altered banding pattern compared to the WT, consistent with exon skipping. (F) Sanger sequencing confirmation of exon skipping. Chromatogram of cDNA obtained from HEK293T cells transfected with the mutant *DNASE2* minigene construct. The sequencing data reveal direct splicing of exon 3 to exon 5, confirming the absence of exon 4 in the mature transcript. (G) Western blot analysis of DNase II protein expression. Western blot analysis of FLAG-tagged DNase II protein products derived from HEK293T cells transfected with wild-type (WT) and mutant (c.511G>A) *DNASE2* minigene constructs. The upper panel shows FLAG-tagged proteins. The lower panel presents tubulin (55 kDa) as a loading control.

To investigate the effect of the identified *DNASE2* variant on splicing, bulk RNA sequencing was performed on P1, her parents, and a healthy control. The patient's mRNA showed no reads mapped to exon 4 of *DNASE2*, with a slight reduction in read count for exon 4 in the parents compared to the healthy control, due to their heterozygous state (Fig. 2A). PCR on cDNA with primers flanking exon 4 revealed distinct banding patterns between P1, her heterozygous parents and healthy control. These results indicate a loss of exon 4 in patient’s mRNA (Fig. 2B). To further elucidate the effect of the splice site variant on DNase II protein production, a minigene analysis was performed in HEK293T cells using both wild-type and mutant constructs carrying the *DNASE2* c.511+5G>A variant (Fig. 2C). PCR with primers flanking exon 4 on cDNA from HEK293T cells transfected with wild-type or mutant constructs showed different sized fragments, indicating a loss of exon 4 in the mutant construct (Fig. 2D). Sanger sequencing confirmed the absence of exon 4 in cDNA generated from HEK293T cells transfected with the mutant construct (Fig. 2E). Western blot analyses using an anti-FLAG antibody demonstrated a significantly smaller protein product in HEK293T cells transfected with the mutant construct. Due to the absence of exon 4 in the DNase II mutant protein, the amino acids 116-170 are lost, consequently reducing the molecular weight of the DNase II mutant protein by approximately 5 kDa (Fig. 2F). DNA degradation assays using patient’s fibroblast and EBV-cell lysates showed reduced levels of DNase II activity against circularized plasmid DNA compared to healthy controls (Fig. 2G). Structural modeling showed that the overall structure of the DNase II homodimer is largely conserved between the wild-type and variant proteins, with most regions aligning closely (Fig. S3). Quantitative comparison revealed an RMSD of 0.594 Å across 276 pruned atom pairs, indicating a high degree of structural similarity in the core region. However, the full alignment across all 305 atom pairs yielded an RMSD of 2.892 Å, reflecting substantial local deviations. The variant protein exhibits significant structural alterations, including the absence of three antiparallel β-sheets and half of an α-helix in the central portion of each monomer. Furthermore, a predicted disulfide bond (Cys19–Cys159) is disrupted by the loss of amino acids 116–170, potentially impairing the stability of the mutant DNase II homodimer.

### d. Immunological Features of the Patients

**Fig. 3:**
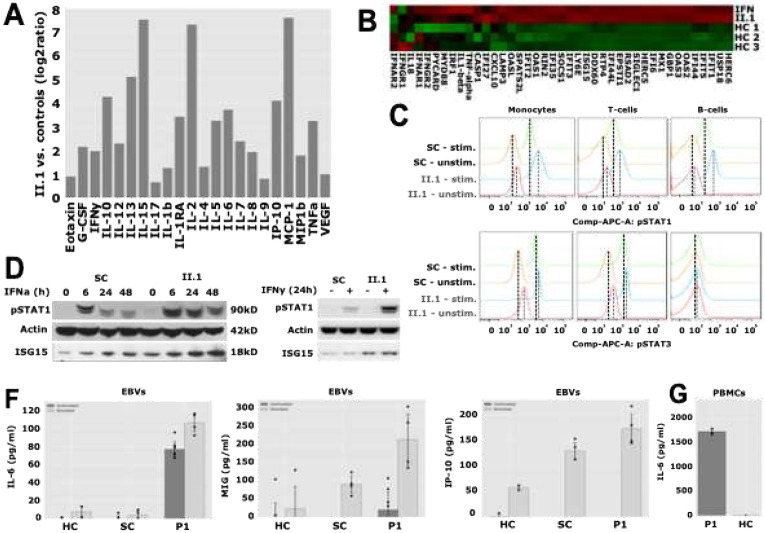
Inflammatory cytokine profile, IFN signature analysis, and immune phenotyping in Patient 1 and controls **(A)** Serum cytokine profiling of P1 compared to family controls. A 27-plex immunoassay was performed, and the most significantly upregulated cytokines are displayed. For each cytokine, the value from P1 was divided by the average of family controls and log_2_-transformed to visualize fold changes. (B) IFN signature analysis by Nanostring. Heatmap displaying the expression levels of IFN-stimulated genes (ISGs) in the patient (II.1) compared to three age-matched healthy controls (HC1-3) and an IFN-positive control (IFN). Red indicates upregulation, while green represents downregulation relative to controls. (C) Phosphorylated STAT (pSTAT) levels in different immune cell populations. Peripheral blood mononuclear cells (PBMCs) from P1 and her sibling control (SC) were stimulated with IFN-α2 (upper panel) or IL-6 (lower panel) for 3 hours. pSTAT1 and pSTAT3 were assessed by flow cytometry using double-staining for pSTATs and cell-type-specific markers (CD14 for monocytes, CD3 for T cells, and CD19 for B cells). Histograms show increased baseline pSTAT1 in P1 and altered response upon IFN-α2 stimulation. IL-6–induced pSTAT3 levels were also measured to evaluate STAT3 pathway activation. (D) Western blot analysis of IFN pathway activation in EBV-transformed B cells. EBV-transformed B cells from P1 and SC were treated with IFN-α2 for the indicated durations. Western blot analysis was performed using specific antibodies against pSTAT1, ISG15, and Actin as a loading control. (E) Sustained IFN response in EBV-transformed B cells. Western blot analysis of EBV-transformed B cells from P1 and SC following 24 hours of IFN-α2 stimulation. The blot shows the expression of pSTAT1, ISG15, and Actin. (F) Cytokine secretion in response to IFN stimulation. ELISA quantification of IP-10 (CXCL10), MIG (CXCL9), and IL-6 levels in culture supernatants from EBV-transformed B cells of a healthy control (HC), SC, and P1 following 24 hours of IFN-γ treatment. Additionally, IL-6 levels were quantified in supernatants from PBMCs of P1 and a healthy control cultured for 48 hours (panel bottom right).

Whole blood cytokine analysis of P1 revealed a markedly elevated inflammatory profile compared to healthy family members, with IL-15, IL-2, and MCP-1 showing the highest fold increases. Importantly, CXCL10, a chemokine strongly induced by type I IFNs, was prominently elevated, alongside IL-6 and TNFα, both implicated in the inflammatory pathology of Dnase II-deficient mouse models (Fig. 3A). Additionally, her CRP and ESR were slightly elevated at the time of sampling. A custom designed NanoString-RNA expression array of 41 IFN-regulated and other inflammatory genes using whole blood from P1 showed moderate to strong upregulation of predominantly IFN-stimulated genes (ISGs) in the patient compared to heathy controls (Fig. 3B). Monocytes, T cells, and B cells isolated from P1 showed constitutively elevated phosphorylated STAT1 (pSTAT1) levels and a hyperresponsive increase upon IFN-α stimulation compared to the unaffected sibling control (SC). Additionally, pSTAT3 levels were already elevated in unstimulated monocytes and T cells from P1, suggesting a baseline activation of STAT3-dependent signaling. In contrast, B cells showed no difference in basal pSTAT3 levels. Upon IL-6 stimulation, a modest but reproducible increase in pSTAT3 was observed in P1’s cells across all lineages, supporting a contribution of IL-6 signaling to the disease pathophysiology (Fig 3C). Western blot of immunoprecipitated extracts from EBV-immortalized lymphoblastic cells of P1 and SC showed constitutively elevated pSTAT1 levels, as well as upon stimulation with IFN-α and IFN-γ. IFN responsive ISG15 levels were also elevated in patients EBV cells (Fig. 3D). ELISA analyses on culture medium obtained from EBV-B cells from P1 showed upregulated cytokine levels, either constitutively (IL-6, 1^st^ panel) or upon stimulation with IFN-γ (MIG and IP-10, 2^nd^ and 3^rd^ panel) compared to controls (Fig. 3F). IL-6 levels were also strongly increased in medium taken from PBMCs from P1 compared to a healthy control (Fig. 3G). Unfortunately, no biospecimen was available for P2.

### e. Functional characterization of variant *USP43*: c.2509G>A; p.E837K

**Fig. 4:**
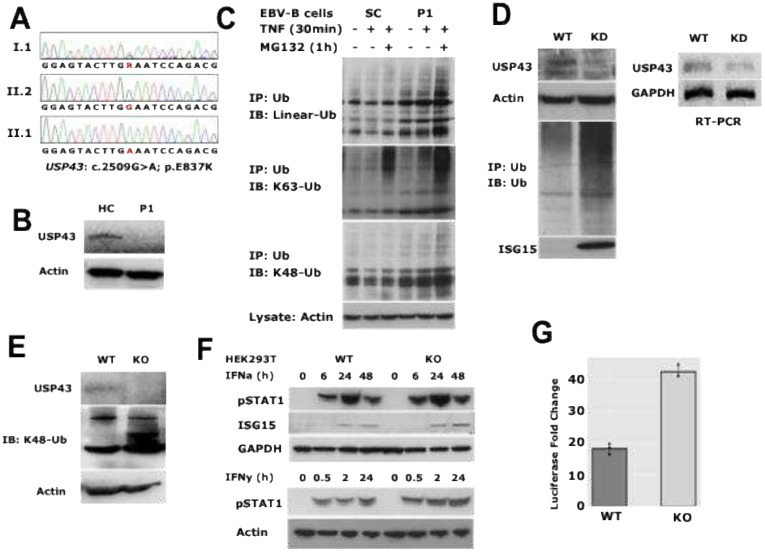
Functional characterization of the USP43 c.2509G>A (p.Glu837Lys) variant in Patient 1 (A) Sanger sequencing analysis of USP43. Chromatogram showing the Sanger sequencing results from DNA extracted from whole blood of Patient 1 (P1), her mother, and her brother, confirming the presence of the homozygous c.2509G>A variant in P1 and heterozygosity in her family members. (B) USP43 protein expression in patient-derived fibroblasts. Western blot analysis of cell lysates from fibroblasts derived from P1 and a healthy control (HC), probed with specific antibodies against USP43. Actin serves as a loading control. A marked reduction in USP43 expression is observed in the patient’s fibroblasts compared to HC. (C) Ubiquitination assay in EBV-B cells. EBV-transformed B cells from P1 and a sibling control (SC) were stimulated with TNF (10 ng/mL) for 30 minutes, the proteasome inhibitor MG132 (10 μM) for 30 minutes, or a combination of both (MG132 added 30 minutes prior to TNF). Immunoprecipitation was performed using ubiquitin (Ub)-specific antibodies, followed by western blotting with chain-specific ubiquitin antibodies. (D) USP43 knockout (USP43KO) generation and validation. Left: HEK293T cells were transfected with a USP43KO CRISPR-Cas9 plasmid along with a homologous recombination-directed repair (HDR) plasmid. After 72 hours, cells were harvested, and immunoprecipitation followed by western blot analysis was performed with indicated antibodies. Right: RT-PCR was conducted on USP43KO cells to assess USP43 transcript levels, confirming efficient knockout. (E) USP43KO stable cell line establishment. HEK293T cells were transfected with a USP43KO nickase plasmid, targeting exon 2 of USP43, and selected with 20 μg/mL puromycin. Single-cell clones were isolated via limiting dilution. Western blot analysis of whole-cell lysates from wild-type (WT) and USP43KO cells was performed using specific antibodies. (F) Impact of USP43 deficiency on IFN signaling. WT 293T and USP43KO cells were treated with 100 ng/mL IFN-α2 or 200 ng/mL IFN-γ for the indicated time points. Cell lysates were analyzed by western blot using antibodies against key IFN-stimulated signaling proteins. (G) WT and USP43KO cells were transfected with an ISRE-driven firefly luciferase reporter plasmid. After 48 hours, cells were stimulated with 100 ng/mL IFN-α2 for 24 hours. Luciferase activity was measured, and fold-change was calculated. Data are representative of at least three independent experiments, demonstrating that USP43 deficiency enhances IFN-stimulated transcriptional activity.

In P1, a homozygous variant in the *USP43* gene (*USP43*: c.2509G>A; p.E837K) was initially identified through a targeted sequencing panel and confirmed by Sanger sequencing, showing segregation within the family (Fig. 4A). Western blot analysis of fibroblast lysates revealed markedly reduced expression of USP43 protein in P1 compared to a healthy control (Fig. 4B). In EBV-transformed B cells from P1, reduced USP43 levels were associated with the accumulation of polyubiquitinated proteins upon stimulation with TNF or TNF plus MG132, compared to a healthy SC (Fig. 4C). This phenotype was further validated in HEK293T cells with transient knockdown of *USP43*, which showed increased global ubiquitination and elevated expression of ISG15, indicating enhanced type I IFN signaling (Fig. 4D). A stable *USP43* knockout (USP43-KO) HEK293T cell line, generated using a CRISPR/Cas9 nickase targeting exon 2 of *USP43*, showed increased levels of K48-linked ubiquitinated proteins under resting conditions (Fig. 4E). Following stimulation with IFN-α or IFN-γ, USP43-KO cells exhibited increased phosphorylation of STAT1 and upregulation of ISG15 compared to wild-type controls (Fig. 4F). Reporter assays using an IFN-stimulated response element (ISRE) luciferase plasmid further confirmed elevated IFN pathway activation in USP43-deficient cells (Fig. 4G). These data demonstrate that loss of USP43 leads to dysregulated protein ubiquitination and heightened IFN signaling. The identified *USP43* variant in P1 likely represents a hypomorphic or LoF allele contributing to the inflammatory phenotype observed in P1.

### f. USP43 reconstitution normalizes ubiquitination, IFN signaling, and inflammatory cytokine production

**Fig. 5:**
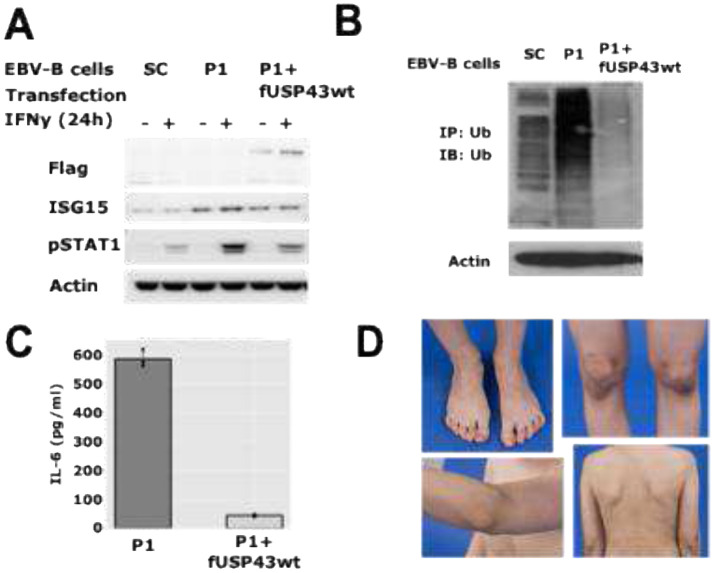
Restoration of IFN signaling and ubiquitination levels by reintroducing wild-type USP43 in USP43-deficient EBV-B cells. (A) Ubiquitination assay in EBV-B cells. EBV-transformed B cells from the sibling control (SC), patient 1 (P1), and patient 1 transfected with a Flag-tagged wild-type USP43 expression construct (P1 + fUSP43wt) were analyzed for ubiquitination levels. Immunoprecipitation (IP) was performed using anti-ubiquitin (Ub) antibodies, followed by western blotting with Ub-specific antibodies to detect total ubiquitination levels. Actin serves as a loading control. Reintroduction of wild-type USP43 rescues the altered ubiquitination pattern observed in P1. (B) Restoration of IFN signaling in USP43-rescued EBV-B cells. EBV-transformed B cells from SC, P1, and P1 transfected with Flag-tagged USP43 (P1 + fUSP43wt) were stimulated with IFN-γ for 24 hours. Western blot analysis of cell lysates was performed using specific antibodies targeting key IFN signaling proteins. The restoration of USP43 expression normalizes IFN-induced signaling responses in patient-derived cells. (C) Normalization of IL-6 secretion upon USP43 rescue. ELISA quantification of IL-6 levels in culture supernatants from EBV-transformed B cells derived from P1, either untransfected or transfected with Flag-tagged USP43 (P1 + fUSP43wt). Reintroduction of wild-type USP43 significantly reduces IL-6 secretion, suggesting its role in modulating inflammatory cytokine production. (D) Clinical improvement in P1 following treatment with ruxolitinib. Post-treatment photographs of patient 1 show complete resolution of all subcutaneous nodules, as well as arthritis and resulting contractures.

Given that loss of USP43 activity led to increased protein ubiquitination and enhanced IFN signaling, we hypothesized that restoring USP43 expression would reverse these abnormalities. To test this, EBV-transformed B cells from P1 were electroporated with a plasmid encoding wild-type, FLAG-tagged USP43, and a stable cell line was established under G418 selection. Immunoblot analysis demonstrated that reconstitution with wild-type USP43 effectively reduced the accumulation of ubiquitinated proteins (Fig. 5A). In parallel, levels of ISG15 and phosphorylated STAT1 (pSTAT1) were decreased in USP43-rescued cells compared to the uncorrected P1-derived EBV-B line (Fig. 5B), indicating normalization of type I IFN signaling. Furthermore, USP43 rescue led to a marked reduction in IL-6 secretion, restoring levels to those observed in healthy control cells (Fig. 5C). These findings support a causal role for USP43 dysfunction in the pro-inflammatory and IFN-related cellular phenotype observed in P1. The patient’s clinical course also reflected the cellular findings. After initiation of treatment with the JAK inhibitor ruxolitinib, P1 experienced a marked improvement in systemic inflammation and growth, eventually achieving long-term clinical remission (Fig. 5D). This supports the pathogenic role of JAK-STAT pathway activation in *DNASE2*/*USP43*-associated autoinflammation.

## Discussion

DNase II deficiency is a rare Mendelian type I interferonopathy characterized by defective lysosomal DNA degradation, resulting in the accumulation of undegraded DNA and persistent immune activation [5]. To date, only 6 patients have been reported, with clinical manifestations predominantly including congenital anemia, hepatosplenomegaly, and systemic inflammation [5,9-11]. Here, we expand the phenotypic spectrum of DNase II deficiency by describing two additional patients with a homozygous splice variant (c.511+5G>A) in *DNASE2*. Our findings highlight additional clinical features, immune abnormalities, and potential genetic modifiers contributing to disease severity (Table1). While previously reported patients share core features such as early-onset cytopenias and recurrent inflammatory fevers, our study broadens the phenotypic spectrum of DNase II deficiency. Notably, both patients presented with progressive joint contractures and features suggestive of osteopenia—skeletal findings not consistently observed in earlier cases—with severe skeletal involvement in P1 [5]. In addition, both patients exhibited prominent skin manifestations, including subcutaneous nodules, rashes, and oral ulcers, which have been only infrequently described in the literature. Consistent with other nucleic acid-driven autoinflammatory disorders, both patients also developed autoantibodies such as anti–double-stranded DNA (anti-dsDNA), ANA, and antiphospholipid antibodies. Anti-dsDNA and related nucleic acid–targeting autoantibodies are a common feature of interferonopathies arising from dysregulated nucleic acid metabolism [24]. Their presence is likely linked to the accumulation of undegraded DNA, which drives chronic activation of nucleic acid-sensing pathways and ultimately leads to a breakdown of self-tolerance [4].

The c.511+5G>A variant in *DNASE2*, identified in both patients, represents the first disease-associated proximal intronic splice site variant linked to DNase II deficiency. Our functional assays demonstrated that this substitution disrupts canonical splicing, resulting in exon 4 skipping and consequent loss of DNase II enzymatic activity. Notably, Rodero et al. previously described a distinct variant (c.347G>C) that also leads to exon 4 skipping, but through a different mechanism. Their variant lies within the coding region of exon 4 and likely disrupts an exonic splice enhancer or the donor site itself, whereas our variant directly affects the conserved intronic splice donor site. These findings show that proximal splice sites play an important role in correct splicing, and that changes in nearby intronic regions can disrupt how exons are recognized. Our results demonstrate that novel prediction tools such as SpliceAI are critical for prioritizing non-coding splice site variants. Subsequent RNA sequencing can subsequently confirm their impact on splicing and support variant reclassification in diagnostic settings [25-28].

Our results further demonstrate that functional validation remains crucial for accurate variant interpretation. The variant c.511+5G>A is currently listed as a variant of uncertain significance (VUS) in ClinVar. Here we provide additional evidence to support its pathogenicity including detection in 2 families and through functional studies [29]. These findings support reclassification of the variant as likely pathogenic or pathogenic and have important implications for diagnostic interpretation and clinical decision-making [30].

Structural modeling of the DNase II mutant protein revealed major alterations resulting from the loss of exon 4, including disruption of a predicted disulfide bond (Cys19–Cys159), which impairs proper folding and dimerization [31]Given the established role of DNase II in lysosomal DNA degradation, we directly tested enzymatic function *in vitro*. DNA degradation assays using lysates from patient-derived fibroblasts and EBV-B cells demonstrated a marked reduction in DNase II activity compared to healthy controls, confirming loss of function. These findings are consistent with prior work by Rodero et al., who similarly showed loss of exon 4 in a different *DNASE2* splice variant associated with impaired nuclease activity [5].

Both patients originated from the same region in Russia - one of Tatar and the other of Bashkir descent - suggesting that the *DNASE2* c.511+5G>A variant may have a higher local frequency in this area. This variant is currently the most frequent pathogenic *DNASE2* variant reported in gnomAD v4.1.0, with a minor allele frequency of 1.8×10^−5^ [32]. The highest frequency is observed in the European (non-Finnish) population, which accounts for 26 out of the 29 heterozygous individuals listed in gnomAD [33]. The identification of this variant in two unrelated individuals from neighboring ethnic groups, despite the absence of known consanguinity in one family, raises the possibility of a regional founder effect. Given the shared Turkic ancestry and close geographic and cultural ties between Tatars and Bashkirs, it is plausible that this variant originated in a common ancestral population [34]. Future population-specific genomic studies are warranted to determine the true carrier frequency in related groups and to further investigate the potential founder origin of this variant.

In P1, we identified an additional novel homozygous missense variant in *USP43* (c.2509G>A; p.E837K), a DUB with emerging roles in immune regulation [19,20,35]. Functional studies revealed that this variant leads to reduced *USP43* expression, increased protein ubiquitination, and an exacerbated type I IFN response. Given that USP43 has been implicated in regulating NF-κB and JAK-STAT signaling, its loss of function could amplify the inflammatory phenotype in DNase II deficiency. The *USP43* gene is tolerant to LoF variants (pLI = 0.01), indicating that heterozygous deficiency is likely benign. However, the homozygous reduction in expression observed in our patient may still contribute to disease. This finding also underscores the broader relevance of digenic and oligogenic inheritance in autoinflammatory and other genetic disorders in founder populations, wherein coexisting variants can act synergistically to exacerbate disease severity, emphasizing the necessity of comprehensive genetic screening to elucidate complex genotype-phenotype relationships [36-40]. Further studies in additional patients and model systems are required to determine whether *USP43* LoF variants consistently act as contributors to interferonopathies.

Our immunological analyses in P1 revealed significantly elevated pro-inflammatory cytokines, with a particularly strong upregulation of type I IFN induced cytokines and IL-6. Mouse models of DNase II deficiency show induction of both IFN-dependent and IFN-independent inflammatory pathways, with TNF-α, IL-1β, and IL-6 implicated in the pathogenesis [6,41]. This supports the idea that cytosolic DNA accumulation triggers a broader inflammatory response beyond type I IFN, mediated in part by cGAS-STING pathway activation. Consistent with this, elevated IL-6 levels were also observed in the patient described by Hong et al., which normalized upon treatment with the JAK inhibitor Baricitinib [10]. In contrast, Rodero et al. reported three patients with DNase II deficiency, but IL-6 levels were only determined in two and found not to be significantly elevated. These interindividual differences may reflect context-specific regulation of cytokine responses. Moreover, given the role of USP43 in ubiquitin-mediated protein degradation, its deficiency may contribute to prolonged IL-6 signaling by impairing the turnover of regulatory proteins involved in cytokine signaling in P1 [42]. These findings raise the possibility that targeting IL-6 signaling may offer a complementary therapeutic avenue beyond type I IFN antagonism, as IL-6 inhibition has shown efficacy in the treatment of other autoinflammatory and immune-mediated diseases [43]. However, further studies involving a larger cohort of patients are needed to validate the relevance and consistency of IL-6 dysregulation in DNase II-associated disease.

Both patients were treated with the JAK1/2 inhibitor ruxolitinib, which led to significant clinical improvement. This included resolution of recurrent fevers and arthritis, reduction in inflammatory markers, improved growth parameters, and stabilization of hepatosplenomegaly. These findings align with previous reports describing the efficacy of JAK inhibitors in treating type I interferonopathies, further supporting their role as a targeted therapeutic strategy for DNase II deficiency [10,44]. Given the persistently elevated JAK-STAT signaling observed in patient-derived immune cells, JAK inhibition represents a rational approach to mitigating the inflammatory burden in this disorder. Future studies should explore long-term outcomes, potential side effects including viral infections and optimal dosing strategies in DNase II-deficient patients undergoing JAK inhibitor therapy.

## Conclusions

This study expands the known phenotypic spectrum of DNase II deficiency, reports a novel pathogenic variant, and suggests that additional genetic modifiers such as USP43 may influence disease severity. Our findings underscore the importance of comprehensive genetic and functional assessments in rare immunological disorders and call for continued surveillance of non-coding variants in diverse populations to refine genotype-phenotype correlations and therapeutic strategies.

## Supplementary Material

This is a list of supplementary files associated with this preprint. Click to download.

• ManuscriptSchnappaufetalSupplememtaryinformation.docx

## Data Availability

The data supporting the findings of this study are available from the corresponding author upon reasonable request.
